# Differential protein expression of DARPP-32 versus Calcineurin in the prefrontal cortex and nucleus accumbens in schizophrenia and bipolar disorder

**DOI:** 10.1038/s41598-019-51456-7

**Published:** 2019-10-16

**Authors:** Yasuto Kunii, Mizuki Hino, Junya Matsumoto, Atsuko Nagaoka, Hiroyuki Nawa, Akiyoshi Kakita, Hiroyasu Akatsu, Yoshio Hashizume, Hirooki Yabe

**Affiliations:** 1 Department of Neuropsychiatry, School of Medicine, Fukushima Medical University, 960-1295 Fukushima, Japan; 2 Department of Psychiatry, Aizu Medical Center, Fukushima Medical University, 969-3492 Fukushima, Japan; 3 Department of Molecular Neurobiology, Brain Research Institute, Niigata University, 951-8585 Niigata, Japan; 4 Department of Pathology, Brain Research Institute, Niigata University, 951-8585 Niigata, Japan; 5 Department of Community-based Medical Education/Department of Community-based Medicine, Nagoya City University Graduate School of Medical Science, 467-8601 Aichi, Japan; 6 Choju Medical Institute, Fukushimura Hospital, 441-8124 Aichi, Japan

**Keywords:** Schizophrenia, Schizophrenia

## Abstract

Dopamine- and cAMP-regulated phosphoprotein of molecular weight 32 kDa (DARPP-32) integrates dopaminergic signaling into that of several other neurotransmitters. Calcineurin (CaN), located downstream of dopaminergic pathways, inactivates DARPP-32 by dephosphorylation. Despite several studies have examined their expression levels of gene and protein in postmortem patients’ brains, they rendered inconsistent results. In this study, protein expression levels of DARPP-32 and CaN were measured by enzyme-linked immunosorbent assay (ELISA) in the prefrontal cortex (PFC), and nucleus accumbens (NAc) of 49 postmortem samples from subjects with schizophrenia, bipolar disorder, and normal controls. We also examined the association between this expression and genetic variants of 8 dopaminergic system-associated molecules for 55 SNPs in the same postmortem samples. In the PFC of patients with schizophrenia, levels of DARPP-32 were significantly decreased, while those of CaN tended to increase. In the NAc, both of DARPP-32 and CaN showed no significant alternations in patients with schizophrenia or bipolar disorder. Further analysis of the correlation of DARPP-32 and CaN expressions, we found that positive correlations in controls and schizophrenia in PFC, and schizophrenia in NAc. In PFC, the expression ratio of DARPP-32/CaN were significantly lower in schizophrenia than controls. We also found that several of the aforementioned SNPs may predict protein expression, one of which was confirmed in a second independent sample set. This differential expression of DARPP-32 and CaN may reflect potential molecular mechanisms underlying the pathogenesis of schizophrenia and bipolar disorder, or differences between these two major psychiatric diseases.

## Introduction

Dopamine- and cAMP-regulated phosphoprotein of molecular weight 32 kDa (DARPP-32), also known as phosphoprotein phosphatase-1 regulatory subunit 1B (PPP1R1B), is a key molecule that integrates dopaminergic signaling into that of several other neurotransmitters, such as a glutamate^1^. Phosphorylated DARPP-32 regulates the physiological activities of various proteins^2^. Calcineurin (CaN) is located downstream of dopaminergic and glutamatergic signaling and inactivates DARPP-32 by dephosphorylation^3^. CaN has also been associated with synaptic plasticity^4^ and neural cell apoptosis^5^. It has been reported that the CaN inhibitor tacrolimus induces psychotic side effects such as hallucinations, anxiety, paranoid delusions, dissociative fugues, and manic-like psychosis^6–8^. Therefore, elucidating the signaling pathway that involves DARPP-32 and CaN may be critical to understanding schizophrenia and other psychiatric disorders.

Several studies have examined the expression of DARPP-32 in the postmortem prefrontal cortex (PFC) of patients with schizophrenia or bipolar disorder^9–14^, but only a few focused on other brain regions^12,13,15–17^. Besides, scarce reports have addressed the protein or mRNA expression of CaN in postmortem brains of patients with schizophrenia^18,19^, and no studies have investigated this expression in patients with bipolar disorder. DARPP-32 and CaN are mostly expressed on dopamine receptor-positive neurons in various brain regions, especially in the caudate, putamen, and nucleus accumbens (NAc)^20,21^. In addition, NAc is an important contributor to the pathogenesis of schizophrenia^22^; it has been especially implicated in generating so-called positive symptoms, such as delusions or hallucinations, on account of its major role in regulating dopamine release and midbrain dopamine system^23^. Nonetheless, the expression of DARPP-32 or CaN in the postmortem NAc of the patients with schizophrenia has not been investigated. Thus, we investigated the expression of DARPP-32 and CaN in NAc and PFC of the patients with schizophrenia.

Meanwhile, numerous studies have attempted, without success, to replicate previously reported genetic associations of multiple candidate genes with schizophrenia. Therefore, in recent years, most researchers have extensively investigated the functional correlations between schizophrenia risk alleles and intermediate phenotypes using neuroimaging, and cognitive and neurophysiologic indexes. Kleinman *et al*. (2011) proposed “genetic neuropathology,” a novel strategy in which the expression of mRNA and proteins in postmortem brains is used as ultimate intermediate phenotypes. They hypothesized that insights into the mechanisms of psychiatric diseases and identification of novel therapeutic targets will emerge from the association of genetic variations with relevant molecular phenotypes in postmortem brains^24^. Actually, several recent postmortem studies^25–28^, including our own^16,29^, have adopted this strategy and provided unique insights into genetic and molecular mechanisms underlying schizophrenia.

Therefore, in this study, we investigated the expression levels of DARPP-32 and CaN proteins in PFC and NAc, regions that receive dopaminergic input and are considered to be affected in schizophrenia. This analysis was performed in 49 postmortem samples from subjects with schizophrenia, bipolar disorder, and control subjects (Table 1) using enzyme-linked immunosorbent assay (ELISA). Moreover, we analyzed the associations between this expression and genetic variants of dopaminergic signaling-associated molecules for 55 single nucleotide polymorphisms (SNPs) in a much larger set of postmortem samples to examine the potential involvement of genetic variants in the pathophysiology of schizophrenia and bipolar disorder.

**Table 1:** Characteristics of postmortem brain samples.

	Schizophrenia	Bipolar Disorder	Control	Cont vs Sz (p value)
**First sample set**				
Number of samples	21	6	22	—
Sex (M/F)	13/8	3/3	14/8	χ^2^-test p = 0.907
Mean age at death (year)	68.9	66.0	63.7	Welch’s *t*-test p = 0.195
Mean PMI (h)	16.1	17.0	14.7	Welch’s *t*-test p = 0.770
Mean CP-eq (mg/day)	653.5	—	—	—
**Second sample set**				
	**Schizophrenia**	**Others**	**Control**	
Number of samples	2	6	26	
Sex (M/F)	1/1	4/2	12/14	
Mean age at death (year)	72	70.8	85.8	
Mean PMI (h)	20.7	4.3	9.6	
Mean CP-eq (mg/day)	1012.5	—	—	

First sample set was used protein expression study in diagnostic groups as well as genotyping. Second sample set was used to confirm the association between rs1801028 and protein expression. PMI, postmortem interval; CP eq, chlorpromazine equivalent dose.

## Results

### Expression of DARPP-32 and Calcineurin in the diagnostic groups

**Figure 1:**
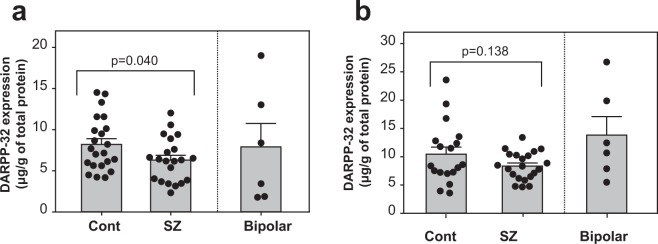
Expression of DARPP-32 in the diagnostic groups. DARPP-32 expression in the BA10 (**a**) and NAc (**b**) of the control group (Cont), the group of patients with bipolar disorder, and the group of patients with schizophrenia (SZ); ELISA was used to measure protein expression. Error bars indicate the standard error of the mean (SEM). p value between controls and schizophrenia was calculated using the Weich’s t-test. Bipolar disorder was shown as a reference.

**Figure 2:**
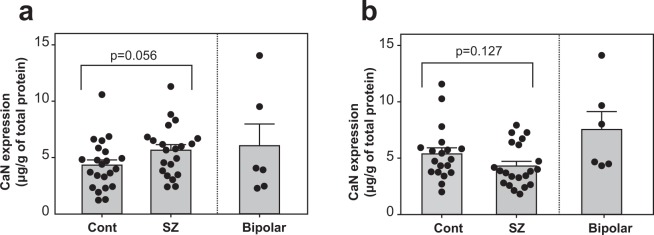
Expression of calcineurin in the diagnostic groups. Calcineurin (CaN) expression in the BA10 (**a**) and NAc (**b**) of the control group (Cont), the group of patients with bipolar disorder, and the group of patients with schizophrenia (SZ); ELISA was used to measure protein expression. Error bars indicate the standard error of the mean (SEM). p value between controls and schizophrenia was calculated using Weich’s t-test. Bipolar disorder was shown as a reference.

In the PFC, we found that the expression levels of DARPP-32 evinced a significant decrease in patients with schizophrenia relative to controls (t (40.14) = 2.12, p = 0.040) (Fig. 1a) and that the expression of CaN tended to increase in patients with schizophrenia (t (40.72) = 1.97, p = 0.056) as compared with controls; the mean of CaN expression in 6 patients with bipolar disorder was higher than that of controls (Fig. 2a). In the NAc, the expression levels of DARPP-32 and CaN showed no significant differences in schizophrenia patients relative to controls (t [23.14] = 1.54, p = 0.138; and t [34.16] = 1.56, p = 0.127; respectively; Figs 1b and 2b); by contrast, each mean of two protein expressions in bipolar patients were higher than that of controls respectively (Figs 1b and 2b).

We also assessed whether the expression level of either DARPP-32 or CaN in patients with schizophrenia was correlated with the daily dosage of antipsychotics that was prescribed in the 3 months immediately preceding each death. There were no significant effects of antipsychotic medication on the expression level of any of these proteins in any brain region examined (all p values > 0.5).

**Figure 3:**
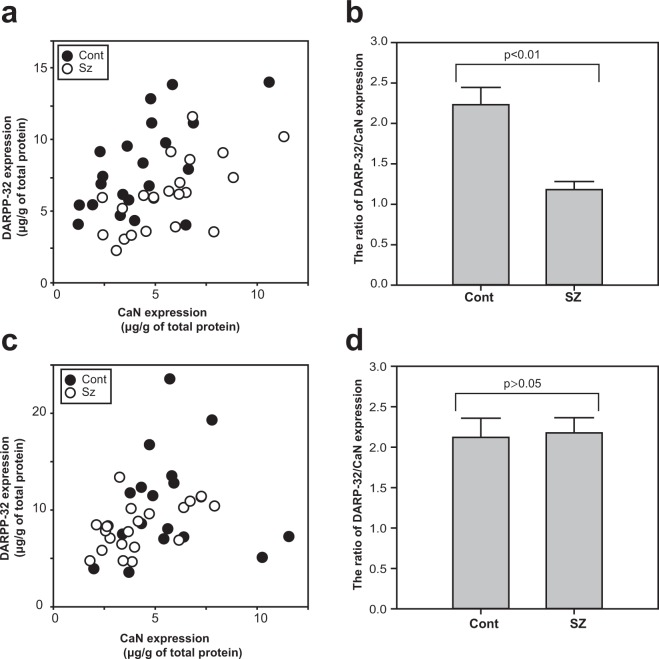
The correlation of the expression levels of DARPP-32 and CaN of control group and patients with schizophrenia. The expression levels of DARPP-32 and CaN in PFC (**a**) and NAc (**c**) of control group (closed circles) and patients with schizophrenia (open circles) are shown by scatter plots. The ratio of DARPP-32 and CaN expression of each sample were compared between diagnostic groups in PFC (**b**) and NAc (**d**). The p values between controls and schizophrenia were calculated using Welch’s t-test. Error bars indicate SEM.

Further we assessed the correlation of expression levels of DARPP-32 and CaN in PFC and NAc (Fig. 3). Positive correlations were observed between the expression levels of DARPP-32 and CaN of controls in PFC (r = 0.585, p = 0.004) (Fig. 3a), patients with schizophrenia in PFC (r = 0.645, p = 0.002) (Fig. 3a) and NAc (r = 0.550, p = 0.010) (Fig. 3c). The ratio of these two proteins in each sample were compared between the diagnostic groups (Fig. 3b,d). In PFC, the ratio of DARPP-32/CaN in each sample were significantly low in schizophrenia patients compared to controls (t [29.72] = 4.45, p = 1.12e-4) (Fig. 3b), but no changes were observed in NAc (t [33.71] = −0.185, p = 0.854) (Fig. 3d).

### Effects of dopaminergic system associated molecules genotype on expression of DARPP-32 and calcineurin

**Figure 4:**
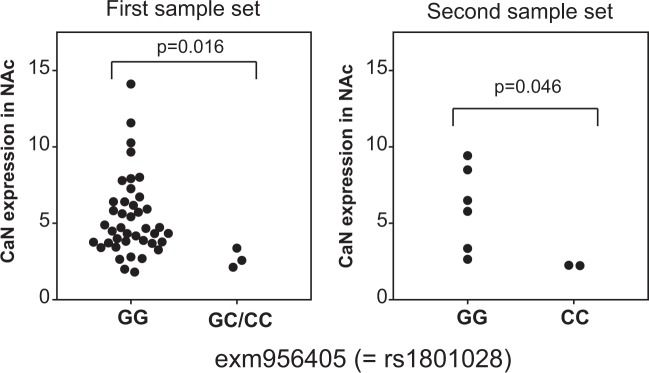
The allelic effect of exm956405 (=rs1801028) on the expression of calcineurin. A significant association was detected between the expression of CaN in the NAc and exm956405. The *p*-value between these groups was calculated using the Mann-Whitney U-test.

We examined the effects of 55 SNPs in 8 dopaminergic system associated molecules (*ANKK1*, *DRD2*, *PPP1R1B*, *PPP3CA*, *PPP3CB*, *PPP3CC*, *PPP3R1*, *and PPP3R2*) on the protein expression levels of DARPP-32 or CaN in a cohort of patients and controls (Table 1) as described in Materials and Methods. The levels of protein expression were compared between minor allele carriers and non-carriers of these SNPs. The results are summarized in Table 2a. Significant associations were observed with 4 SNPs in the *DRD2* (2), *PPP3CB* (1), and *PPP3CC* (1). These SNPs predicted protein expression (DARPP-32 or CaN) in the postmortem brains (BA10 or NAc). As shown in Table 2b, analysis of a second independent sample set confirmed only 1 SNPs out of 4SNPs: exm956405 (=rs1801028). There was a significant association between the expressions of CaN in the NAc and exm956405 (Fig. 4; first sample set: U = 10.0, p = 0.016; second sample set: U = 0.0, p = 0.046).

**Table 2:** Summary of allelic effects of SNPs of dopaminergic-system associated molecules on DARPP-32 and calcineurin expression.

Gene	SNP	exonic/intronic, and substitution	Protein	Region	The numbers of minor-allele carriers and non-carriers	Hardy–Weinberg Equilibrium (χ2, P value)	The ratios of protein expression between minor-allele carriers and non-carriers		p value (Mann-Whitney)
**(a) First sample set**									
*DRD2*	exm956405 (=rs1801028)	exonic, Ser ⇒ Cys	CaN	NAc	3/42	(10.2, 0.001)	0.55	(C+/C−)	0.016
*DRD2*	exm956405 (=rs1801028)	exonic, Ser ⇒ Cys	CaN	BA10	3/45	(11.0, 0.001)	1.78	(C+/C−)	0.023
*DRD2*	rs2734833	intronic	DARPP	NAc	3/41	(44.0, 0)	0.67	(T+/T−)	0.043
*PPP3CB*	exm2259632 (=rs 12644)	exonic	DARPP	BA10	34/14	(1.92, 0.166)	1.35	(T+/T−)	0.015
*PPP3CC*	rs1116084	intronic	DARPP	BA10	37/11	(0.756, 0.385)	1.68	(A+/A−)	0.016
**(b) Second sample set**									
**Gene**	**SNP**	**exonic/intronic, and substitution**	**Protein**	**Region**			**The ratios of protein expression between minor-allele carriers and non-carriers**		**p value (Mann-Whitney)**
*DRD2*	exm956405 (=rs1801028)	exonic, Ser ⇒ Cys	CaN	NAc	2/6	(8, 0.0047)	0.37	(C+/C−)	0.046

## Discussion

Our study is the first analysis of DARPP-32 protein expression levels in the NAc of the patients with schizophrenia and those of CaN in the postmortem brain tissue of the patients with bipolar disorder. We showed that the levels of DARPP-32 in the PFC of the patients with schizophrenia significantly decreased, while those of CaN tended to increase in the PFC of the patients with schizophrenia. On the other hand, levels of DARPP-32 and CaN showed no significant differences in the NAc of the patients with schizophrenia or bipolar disorder. Moreover, in the PFC we found that positive correlations between expressions of DARPP-32 and CaN and the expression ratio of DARPP-32/CaN were significantly lower in schizophrenia than controls. As aforementioned, the expression of DARPP-32 in the postmortem brain of patients with schizophrenia or bipolar disorder has been largely investigated. While the levels of DARPP-32 protein in the PFC were decreased in patients with schizophrenia and bipolar disorder^9,11,12^, findings relevant to the mRNA levels in these regions were inconsistent across studies^10,13,14,30,31^. Alternatively, a few studies have investigated postmortem expression of CaN in the brains of the patients with schizophrenia^18,19,32–34^, but no studies have analyzed this expression in bipolar disorder. These postmortem studies of DARPP-32 or CaN in schizophrenia and/or bipolar disorder are summarized in Table 3. Anyway, the most interesting findings in this study was that in the PFC there were positive correlations between DARPP-32 and CaN expression, and lower DARPP-32/CaN ratio in schizophrenia, which were firstly reported to the best of our knowledge. We could point out several suggestions from these results: (1) DARPP-32 and CaN were usually coexistent in the brain, even if in schizophrenia, (2) the balance between DARPP-32 and CaN was greatly disrupted in schizophrenia, and (3) these ware consistent with the results of the above previous postmortem studies review.

**Table 3:** Overview of postmortem brain studies focused on DARPP-32(a) and CaN(b).

Number of resources	Brain Region	Taget molecule	Method	Main findings	Refference
**(a) Postmortem brain studies of DARPP-32**					
SZ(14), CONT(14)	PFC	Protein	Immunoblot	DARPP-32 expression was reduced in SZ	Albert *et al*.^9^
SZ(12), BP(10), CONT(11)	PFC	Protein	IHC, Immunoblot	DARPP-32 expression was lower in SZ and BP	Ishikawa *et al*.^11^
SZ(9), CONT(9)	PFC, Hip, Striatum	Protein	IHC	DARPP-32 immnoreactive neurons were lower in layers II-V of PFC in SZ	Kunii *et al*.^12^
SZ(11), CONT(10)	Insula cortex	Protein	IHC	DARPP-32 immnoreactive neurons were lower in layers II-III of inslula in SZ	Nishiura *et al*.^17^
SZ(11), CONT(11)	STG	Protein	IHC	DARPP-32 immnoreactive neurons were lower in layers III-IV of STG in SZ	Kunii *et al*.^15^
SZ(12), CONT(12)	Striatum	Protein	Immunoblot	DARPP-32 expression was increased in CAU of SZ	Kunii *et al*.^16^
SZ(13), CONT(8)	Thalamus	mRNA	ISH	No chnages in DARPP-32 mRNA expression in thalamic nuclei of SZ	Clinton *et al*.^31^
SZ(18), CONT(11)	PFC, ACC	mRNA	ISH	No chnages in DARPP-32 mRNA expression in PFC and ACC of SZ	Baracskay *et al*.^10^
SZ(35), BP(35), CONT(35)	PFC	mRNA	Realtime PCR	DARPP-32 mRNA expression was reduced in PFC of SZ who died by suicide	Feldcamp *et al*.^30^
SZ(33), BP(32), CONT(34)	PFC	mRNA	Realtime PCR	DARPP-32 mRNA expression was increased in PFC of SZ and BP	Zhan *et al*.^14^
SZ (n = 176), BP (n = 61), MDD(138)CONT (n = 283)	PFC, Hip, Cau	mRNA	Realtime PCR	t-DARPP mRNA expression was increased in PFC of SZ and BP	Kunii *et al*.^13^
**(b) Postmortem brain studies of CaN**					
**Number of resources**	**Brain Region**	**Taget molecule**	**Method**	**Main findings**	**Refference**
SZ(15), CONT(15)	PFC, Hip	Protein	Immunoblot	No chnages in CaN in PFC and Hip of SZ	Kozlovsky *et al*.^19^
SZ(9), CONT(9)	PFC, Hip, Striatum	Protein	IHC	CaN immnoreactive neurons were increased in caudate of SZ	Wada *et al*.^33^
SZ(12), CONT(12)	Striatum	Protein	Immunoblot	No chnages in CaN in caudate and putamen of SZ	Kunii *et al*.^16^
SZ(11), CONT(11)	STG	Protein	IHC	CaN immnoreactive neurons were increased in layers II-VI of STG in SZ	Wada *et al*.^34^
SZ(12), CONT(12)	PFC	mRNA	DNA microarray (cDNA)	CaN A mRNA was increased in PFC of SZ	Hakak *et al*.^32^
SZ(13), CONT(12)	Hip	Protein, mRNA	ELISA, RT-PCR	CaN A and mRNA were decreased in hip of SZ	Eastwood *et al*.^18^

SZ, schizophrenia; CONT, control; BP, bipolar; PFC, prefrontal cortex; Hip, hippocampus; STG, superior temporal gyrus; ACC, anterior cingulate gyrus; Cau, caudate; IHC, immunohistochemistry; ISH, *in situ* hybridization; ELISA, enzyme-linked immunosorbent assay; CaN, calcineurin.

If decreased DARPP-32/CaN expression ratio is the primary pathophysiological alteration in the patients with schizophrenia, that means that upregulation of CaN promotes the inactivation of DARPP-32^35^. Decreased DARPP-32 activity induces a reduction in dopaminergic signaling in the PFC, which results in a so-called “hypofrontality” in schizophrenia. It is possible that a decrease in DARPP-32 expression in the PFC observed both in the current as well as in previous studies results, at least in part, from this process. On the contrary, it is of interest that the changes in CaN expression in the NAc evinced opposite tendencies in patients with schizophrenia and those with bipolar disorder. Since, upon the stimulation of dopamine D_1_ receptor, CaN inactivates DARPP-32 by dephosphorylation, it can be concluded that CaN exerts a buffering action against excessive dopamine signaling. It is probable that variations in CaN expression may reflect different dopaminergic pathologies between these two major psychiatric diseases.

We also found that several SNPs of the gene encoding DRD2 and CaN were associated with DARPP-32 or CaN protein expression in postmortem brains. In particular, the polymorphism of protein phosphatase 3 catalytic subunit gamma (*PPP3CC*), a gene encoding the CaN γ-catalytic subunit, was previously found to be relevant to schizophrenia. Genetic studies revealed a significant association between genetic variations of *PPP3CC*^36^ or *PPP3CC* gene haplotype^37^ and the incidence of schizophrenia; however, no correlations were found for other population samples^38–40^. In addition, an association between the *PPP3CC* gene and bipolar disorder was also reported^41^. Another study using forebrain-specific *PPP3CC* gene-knockout mice reported multiple abnormal behaviors associated with schizophrenia, such as decreased social interaction and impairments in prepulse inhibition^42^.

**Figure 5:**
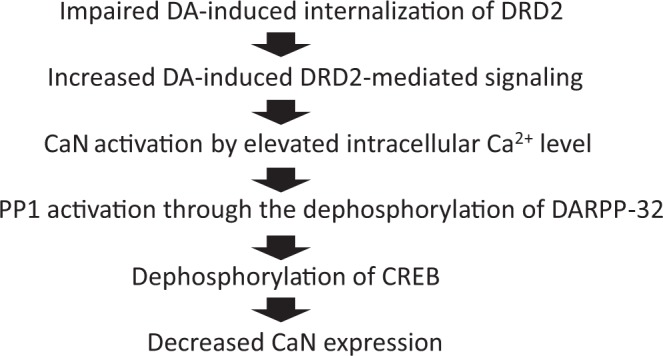
Flow chart of the DARPP-32-CaN pathway in the Cys311 variant of DRD2. The disturbance of DRD2 desensitization can prolong dopamine-induced DRD2-mediated signaling and accelerate the following processes; PLC activation, IP3 release, and Ca^2+^ release from the endoplasmic reticulum. As a result, increased intracellular Ca^2+^ activates CaN. The activated CaN can then promote PP1 activation through the dephosphorylation of DARPP-32. Dephosphorylation of CREB may then be promoted in the brains carrying the Cys311 variant of DRD2. Since the ChIP analysis showed that CREB binds upstream of several CaN genes, dephosphorylation of CREB may decrease CaN expression. DRD2, dopamine D2 receptor; PLC, phospholipase C; IP3, Inositol trisphosphate; CaN, calcineurin;PPI,;CREB, cAMP response element-binding protein; ChIP, Chromatin immunoprecipitation.

In a second independent sample set, we could confirm only 1 SNP, exm956405 (=rs1801028), that predicted CaN expression in the NAc. This SNP was identified as a novel missense nucleotide change causing an amino acid substitution of serine with cysteine at codon 311 (Ser311Cys) in *DRD2*^43^. An association study showed that Cys311 in *DRD2* was significantly associated with schizophrenia^44^. Moreover, clinical assessment found that patients with schizophrenia that exhibited the Cys311 allele spent less time sin the hospital, presented less severe negative symptoms, and were more often married compared to patients without Cys311^44^. These results suggest that patients with schizophrenia that have the Cys311 allele may respond well to antipsychotic treatment and, as a result, shows relatively mild negative symptoms. A several meta-analyses also demonstrated this association^45–47^. The tendency of this association was observed in our samples of this study (relative risk; 2.968, Fisher’s exact test; p = 0.071). It was further demonstrated that dopamine-induced internalization of DRD2 was impaired in the Cys311 variant of DRD2^48^. Since the internalization of DRD2 contributes to its desensitization^49^, the desensitization of DRD2 may also be suppressed in patients with the Cys311 variant. Thus, the disturbance of DRD2 desensitization can prolong dopamine-induced DRD2-mediated signaling and accelerate the subsequent processes (Fig. 5). Eventually, dephosphorylation of cAMP response element-binding protein (CREB) binds upstream of four CaN genes (*PPP3CA*, *PPP3CB*, *PPP3CC*, *PPP3R1*; found through the ChIP-Atlas database at “chip-atlas.org”) and then decreases CaN expression. This assumed mechanism (Fig. 5) was consistent with our finding that subjects with Cys311 in *DRD2* featured lower levels of CaN in the NAc (Fig. 4). In other words, the C allele of rs1801028 was at risk for schizophrenia and was associated with lower CaN expression in the NAc.

The present study performed on brain samples has several limitations that require further attention. First, confounding effects of disease-related factors including medication may have affected the expression of DARPP-32, CaN, or both. Although we did not observe any effects of antipsychotic drugs on the levels of DARPP-32 or CaN expression in this study, additional animal studies are necessary to investigate the effects of chronic antipsychotic administration on the expression of these proteins in the PFC and NAc. Second, our study population was relatively small, especially for a genetic study. In addition, we could not uniformly sample from group with sufficient equilibrium because the NAc was a relatively small area and there were a small number of NAc available. As a result, several genotypes in this study including rs1801028 were not two in Hardy–Weinberg Equilibrium. The findings must be, therefore, confirmed via postmortem examination of a larger brain cohort.

In conclusion, our results show that the expressions of the DARPP-32 and CaN protein were altered in postmortem brains of the patients with schizophrenia and bipolar disorder compared to those from control subjects, which is accompanied by genetic variations. Our results may reflect potential molecular mechanisms underlying the pathogenesis of schizophrenia and bipolar disorder or differences between these two major psychiatric diseases.

## Materials and Methods

### Human postmortem brain tissue collection

Postmortem brain tissue samples from patients with schizophrenia, bipolar disorder, and control subjects were obtained from Fukushima Brain Bank at the Department of Neuropsychiatry, Fukushima Medical University; Brain Research Institute, Niigata University; and Choju Medical Institute Fukushimura Hospital, Toyohashi as described previously^29^. Use of postmortem human brain tissues for the present study was approved by the Ethics Committee of Fukushima Medical University and Niigata University, and complied with the Declaration of Helsinki and its later amendments. All procedures were carried out with the informed written consent of the next of kin. Detailed demographic information of brain tissues from 21 subjects with schizophrenia, 6 subjects with bipolar disorder and 22 control subjects used in this study (it was identical to first sample set in genotyping) was summarized in Table 1. Each patient with schizophrenia and bipolar disorder fulfilled the diagnostic criteria established by the American Psychiatric Association (Diagnostic and Statistical Manual of Mental Disorders: DSM-IV). For patients with schizophrenia, the daily dosage of antipsychotics prescribed during the 3 months immediately preceding death is shown as chlorpromazine-equivalent dose (mg/day, Table 1).

### DNA collection and SNP genotyping

The subjects for the association analysis between SNPs and protein expression consisted of 27 patients (16 men; mean age, 68.2 ± 15.2 years) and 22 control subjects (14 men; mean age, 63.7 ± 14.4 years). A second replication sample set consisted of 34 subjects (17 men; mean age, 82.4 ± 9.4 years). Genomic DNA was extracted from the frozen cerebellum or occipital cortex and genotyping was performed using HumanCoreExome −24 v1.0 Beadchip on an iScan system (Illumina, Tokyo, Japan) as described previously^29^. Of 128 SNPs derived from 8 dopaminergic system-associated genes [Ankyrin repeat and kinase domain containing 1(ANKK1), dopamine D2 receptor (DRD2), phosphoprotein phosphatase-1 regulatory subunit 1B(PPP1R1B), protein phosphatase 3 catalytic subunit alpha (PPP3CA), protein phosphatase 3 catalytic subunit beta (PPP3CB), protein phosphatase 3 catalytic subunit gamma (PPP3CC), protein phosphatase 3 regulatory subunit B alpha (PPP3R1), and protein phosphatase 3 regulatory subunit B beta (PPP3R2)] included in the chip, we excluded SNPs with call rates of <95% and minor allele frequencies of <1%; the number of SNPs analyzed thus totaled to 55 (Supplementary Table S1).

### Tissue retrieval and processing for ELISA

Pieces (weighing approximately 100 mg) of gray matter tissue from Brodmann area 10 (BA10) as PFC and NAc were isolated from frozen brain. For PFC, gray matter tissue was dissected from the frontal pole. NAc was identified on the frozen coronal slabs corresponding to the anterior one-third of the inferior temporal cortex. These frozen brain tissues were suspended in the 2% SDS solution, incubated for 20 min, subjected to three freeze-thaw cycles, and sonicated for 10 min. Then, the samples were diluted in phosphate buffered saline (PBS; 137 mM NaCl, 2.7 mM KCl, 10 mM Na_2_HPO_4_, 1.76 mM KH_2_PO_4_) so that the final concentration of SDS was below 0.2%. The expression of proteins was determined by using commercial ELISA kits (SEB323Hu for Calcineurin, and SEE017Hu for DARPP-32, Cloud Clone Corp, Houston, TX). The analysis was performed according to the manufacturer’s protocols. The expression levels of each protein were normalized against the total protein concentration.

### Statistical analysis

Demographic variables (sex, age, and PMI) were compared between the groups using a χ^2^-test and Welch’s t-test. The latter was also used for comparing the levels of protein (DARPP-32, CaN) expression between the diagnostic groups (schizophrenia and control, with bipolar disorder as a reference). The correlations of expression levels of DARPP-32 and CaN in each sample were assessed by Pearson’s correlation coefficients, and the ratio of proteins were compared between the diagnostic groups by Welch’s t-test. For association study of SNPs, we divided all samples into minor allele carriers and non-carriers of each SNPs. A Mann-Whitney *U*-test was then used for comparing the levels of protein expression between the genotypes of each SNP. In the comparison between the two groups, we excluded outliers (>mean ± 3 SD) from analysis. Further, Spearman’s rank correlation coefficients were used to assess the association between the expressions of each protein and estimated total dosage of neuroleptic drugs. SPSS ver. 25.0 (SPSS, Chicago, IL, USA) and (SigmaPlot14.0; Systat Software Inc., San Jose, CA, USA) was used for analysis.

## Supplementary information

Table legends

Supplementary Table S1
