# Supplementary material for: Differential protein expression of DARPP-32 versus Calcineurin in the prefrontal cortex and nucleus accumbens in schizophrenia and bipolar disorder
Source: Sci Rep. 2019 Oct 16;9:14877. doi: 10.1038/s41598-019-51456-7 (PMC6796065; doi:10.1038/s41598-019-51456-7)
Supplement: Supplementary file 1 — Table legends [file 41598_2019_51456_MOESM1_ESM.docx]

**Supplementary table legends**

**Supplementary Table S1. A list of candidate SNPs tested for association with expression of DARPP-32 and calcineurin.**

Genotyping of 55 SNPs in the dopaminergic system associated molecules [Ankyrin repeat and kinase domain containing 1(*ANKK1*), dopamine D2 receptor (*DRD2*), phosphoprotein phosphatase-1 regulatory subunit 1B(*PPP1R1B*), protein phosphatase 3 catalytic subunit alpha(*PPP3CA*), protein phosphatase 3 catalytic subunit beta (*PPP3CB*), protein phosphatase 3 catalytic subunit gamma (*PPP3CC*), protein phosphatase 3 regulatory subunit B alpha(*PPP3R1*), and protein phosphatase 3 regulatory subunit B beta (*PPP3R2*)] was performed using HumanCoreExome -24 v1.0 Beadchip on an iScan system.
